# Antipredator phenotype in crucian carp altered by a psychoactive drug

**DOI:** 10.1002/ece3.7762

**Published:** 2021-06-14

**Authors:** Jerker Vinterstare, Christer Brönmark, P. Anders Nilsson, R. Brian Langerhans, Olof Berglund, Jennie Örjes, Tomas Brodin, Jerker Fick, Kaj Hulthén

**Affiliations:** ^1^ Department of Biology Aquatic Ecology Unit, Ecology Building Lund University Lund Sweden; ^2^ Department of Biological Sciences and W.M. Keck Center for Behavioral Biology North Carolina State University Raleigh NC USA; ^3^ Department of Wildlife, Fish and Environmental Studies Swedish University of Agricultural Sciences (SLU) – Umeå Umeå Sweden; ^4^ Department of Chemistry Umeå University Umeå Sweden

**Keywords:** antipredator traits, inducible defenses, phenotypic plasticity, psychoactive drugs, serotonergic system, SSRI

## Abstract

Predator‐inducible defenses constitute a widespread form of adaptive phenotypic plasticity, and such defenses have recently been suggested linked with the neuroendocrine system. The neuroendocrine system is a target of endocrine disruptors, such as psychoactive pharmaceuticals, which are common aquatic contaminants. We hypothesized that exposure to an antidepressant pollutant, fluoxetine, influences the physiological stress response in our model species, crucian carp, affecting its behavioral and morphological responses to predation threat. We examined short‐ and long‐term effects of fluoxetine and predator exposure on behavior and morphology in crucian carp. Seventeen days of exposure to a high dose of fluoxetine (100 µg/L) resulted in a shyer phenotype, regardless of the presence/absence of a pike predator, but this effect disappeared after long‐term exposure. Fluoxetine effects on morphological plasticity were context‐dependent as a low dose (1 µg/L) only influenced crucian carp body shape in pike presence. A high dose of fluoxetine strongly influenced body shape regardless of predator treatment. Our results highlight that environmental pollution by pharmaceuticals could disrupt physiological regulation of ecologically important inducible defenses.

## Graphical abstract

We examined short‐ and long‐term effects of fluoxetine on the anti‐predator phenotype in crucian carp. We found effects of fluoxetine on both behaviour and body shape, highlighting both proximate mechanism behind external defence regulation but also that environmental pollution by pharmaceuticals could disrupt physiological regulation of ecologically important inducible defences.

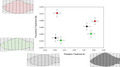

## 1 INTRODUCTION

Predation is an important structuring force in nature and has selected for the evolution of diverse defense adaptations in prey organisms, including behavioral (Sih, 1980, Ydenberg & Dill, 1986), morphological (Brönmark & Miner, 1992; Young et al., 2004) and chemical defenses (Bakus, 1981, Brodie Jr, 1977, Skelhorn & Rowe, 2005). Antipredator defenses can be constitutive, that is, always expressed by the host regardless of the prevailing risk of predation. Many prey organisms however confront intermittent and unpredictable predation risk, which may instead favor phenotypically plastic defense traits, that is, the ability to express adaptive phenotypes according to prevailing risk of predation (DeWitt & Scheiner, 2004). In spite of recent progress in our theoretical and empirical understanding of the ecology and evolution of phenotypic plasticity in antipredator traits (e.g., Weiss et al., 2015, Creel, 2018, Mitchell et al., 2017), the proximate, physiological mechanisms behind inducible defenses, behavioral as well as morphological, remain elusive.

Contemporary studies highlight that inducible defense expression may be mediated by physiological stress response mechanisms, specifically predator‐induced activation of the hypothalamus–pituitary–adrenal/interrenal axis (HPA/HPI axis) and associated hormones (mainly glucocorticoids) (Hossie et al., 2010; Maher et al., 2013; Vinterstare et al., 2020). In aquatic environments, chemical cues produced by predators or conspecifics can activate the neuroendocrine stress axis, which in turn may alter glucocorticoid levels (e.g., cortisol) in for example fish (Oliveira et al., 2014; Rehnberg et al., 1987) and tadpoles (Fraker et al., 2009). Furthermore, direct experimental manipulations of the stress axis have unraveled links between glucocorticoid concentrations and tadpole tail morphology, an inducible defense trait (Maher et al., 2013). Cortisol is also a key hormone in mediating the expression of behavioral responses to predation threat (Sapolsky et al., 2000). Moreover, consistent differences in individual behavior (animal personality, stress‐coping styles, (Koolhaas et al., 2010; Reale et al., 2010; Sih et al., 2004)) are suggested associated with individual differences in stress‐specific physiological responses (Koolhaas et al., 2010). Hence, physiology, neuroendocrine pathways, and behavioral and morphological responses to predation threat appear causally interrelated (Maher et al., 2013).

A corollary of recent research suggests a properly functioning neuroendocrine system, a prerequisite for appropriate induction of plastic antipredator defenses. However, the neuroendocrine system is a target of endocrine disruptors, including environmental contaminants such as pharmaceuticals (e.g., Sehonova et al., 2018). Numerous studies highlight that psychoactive pharmaceuticals enter waterways via treated wastewater effluents and remain biochemically active in aquatic environments (e.g., Brodin et al., 2013; Muir et al., 2017). These pharmaceuticals are explicitly designed to modulate human physiology and behavior, and may remain therapeutically active at low concentrations (Vaswani et al., 2003). Receptors and physiological pathways are evolutionary conserved in vertebrates, and thus, it is highly likely that pharmaceuticals affect also nontarget aquatic organisms (Fent et al., 2006; Sehonova et al., 2018). However, we still know very little about how pharmaceuticals affect aquatic wildlife and predator–prey interactions.

One class of pharmaceuticals of special concern is the selective serotonin reuptake inhibitors (SSRIs), such as the commonly prescribed antidepressant fluoxetine (marketed as Prozac^TM^), that are commonly detected in aquatic environments (e.g., Weinberger & Klaper, 2014 and references therein, Mole & Brooks, 2019). SSRIs alter the serotonergic system by inhibiting serotonin reuptake mechanisms, increasing extracellular concentration of serotonin within the central nervous system (Vaswani et al., 2003). The serotonergic system plays a key role in several biological functions, including effects on phenotypic traits such as behavior and physiology of individuals (Winberg & Thörnqvist, 2016, Lucki, 1998, Koolhaas et al., 2007, Backström & Winberg, 2017, Koolhaas et al., 2010). However, due to the complexity of the system we lack a unifying physiological function for serotonin (Fischer & Ullsperger, 2017). It is nevertheless agreed that serotonin plays a key role in the stress response by modulating the HPA/HPI axis and production and secretion of glucocorticoids (Lowry, 2002, Gesto et al., 2013; Lepage et al., 2005; Hesketh et al., 2005). Hence, exposure to fluoxetine can impact organismal responses to various stressors, including predation threat. However, there is no consensus on how fluoxetine/SSRI influences antipredator traits in prey organisms. For example, some studies report increased activity and reduced antipredator behavior upon fluoxetine exposure, whereas other show opposite trends (Martin et al., 2017, Abreu et al., 2017, Barry, 2013, Saaristo et al., 2017). Moreover, temporal aspects, that is, the definition of ecologically relevant exposure periods, vary in the literature. Currently, the literature is heavily biased toward short‐term, acute exposure studies (hours to days) where trait responses may differ from the long‐term, chronic exposure organisms experience under natural conditions (Herculano & Maximino, 2014; Martin et al., 2017; McCallum et al., 2017). For example, studies have shown that serotonin stimulates the release of glucocorticoids during short‐term SSRI exposure, whereas under chronic exposure an increase in serotonin levels is linked to reduced stress sensitivity, that is, diametrically opposite effects (Vera‐Chang et al., 2018; Winberg & Thörnqvist, 2016).

Here, we quantify morphological and behavioral responses in crucian carp (*Carassius carassius*) following manipulations of perceived predation risk (presence/absence of pike, *Esox lucius*) in combination with experimental exposure to fluoxetine. Crucian carp, a widely distributed Eurasian freshwater fish, provided the first example of a predator‐induced morphological defense in a vertebrate: induction of increased body depth in the presence of predatory fish (Brönmark & Miner, 1992). Increased body depth efficiently reduces predation rates from gape‐limited predators, such as pike (Nilsson et al., 1995), and enhances fast‐start escape performance (Domenici et al., 2008). The morphological defense is induced by chemical cues associated with the diet of their natural predators (Brönmark & Pettersson, 1994). Chemical cues of predators also affect behavioral traits in crucian carp (Pettersson et al., 2000; Ranåker et al., 2012). Furthermore, the main glucocorticoid in fish, that is, cortisol, appears to be involved in the proximate physiological mechanism driving morphological defense expression, as in amphibian tadpoles (Maher et al., 2013). Specifically, recent work found a strong effect of endogenous cortisol implants on the expression of predator‐induced traits (body depth and body coloration) in crucian carp (Vinterstare, Hulthén, Nilsson, Nilsson Sköld & Brönmark, 2020), implying that stress physiology and defense expression are linked via the HPA/HPI axis (Sapolsky et al., 2000).

In this study, we test the link between stress physiology and defense adaptations in crucian carp. We conducted a long‐term factorial experiment where wild‐caught and predator‐naïve crucian carp were exposed to different concentrations of fluoxetine and reared in the presence or absence of predatory pike. We hypothesized that exposure to fluoxetine would influence the physiological stress response in crucian carp, in turn affecting the behavioral and morphological responses to predation threat. We predicted that fluoxetine exposure would induce a shyer stress‐coping style after short‐term exposure, whereas long‐term exposure would result in acclimation of the serotonergic system, causing reduced stress sensitivity. Based on earlier studies (Maher et al., 2013; Vinterstare, Hulthén, Nilsson, Nilsson Sköld & Brönmark, 2020), we predicted that chronic fluoxetine exposure would reduce the magnitude of morphological defense expression in the presence of predator cues.

## 2 MATERIAL AND METHODS

### 2.1 Study animals

We collected crucian carp with dip nets in lake Trollsjön, southern Sweden (55°50′15.3′′ N, 13°17′16.4′′ E) 20–22 April 2015. Trollsjön are known to be free from piscivorous predators, and hence, all crucian carp were predator‐naïve and expressed the nondefended phenotype (shallow body depth). After capture, individuals (*n* = 144, total length: 11.56 ± 0.03 cm; mean ± *SE*) were immediately transported to experimental facilities at Lund University. Fish were individually tagged by surgically implanting a Passive Integrated Transponder (PIT) tag (Texas Instruments, USA; half‐duplex, 134 kHz, 23.1 mm long, 3.85 mm diameter, 0.6 g in air) into the coelomic cavity of the fish (Skov et al., 2005), after which they were acclimated for four weeks in a filtered and aerated 385 L tank. Food (fish pellets, frozen shrimp mix) was supplied ad libitum during the acclimation period. Pike (*n* = 12, size range 37–42 cm) were electrofished in lake Krankesjön, southern Sweden, and held individually on a crucian carp diet for at least eight weeks prior to experiments. The study was performed under permission from the Lund/Malmö authority for ethics of animal experimentation (license M36‐14).

### 2.2 Experimental treatments

To assess effects of fluoxetine and the presence/absence of a predator on crucian carp behavior and body morphology, we performed a 2 × 3 factorial experiment with the factors “Predator” (pike presence/absence) and fluoxetine, from now on referred to as “FLX” (No, Low FLX and High FLX concentration). We set up 24 aquaria (152 L; 95 × 40 × 40 cm) kept at 18ºC and 12:12 light:darkness. Each aquarium was divided into two equally sized compartments by a transparent, perforated acrylic partition. Crucian carp in groups of six were haphazardly assigned to one of the compartments of each aquarium, and a single pike was assigned to the other compartment in the predator treatment, whereas in controls it remained empty. The transparent, perforated partition dividing experimental tanks allowed water circulation between compartments, so crucian carp could detect pike by visual and chemical cues. Three sides of each aquarium were externally covered with black plastic film to prevent visual interactions between aquaria. Each aquarium was continuously aerated. Treatments were assigned to aquaria by random permutation and all treatments were replicated four times.

FLX(CAS# 59333‐67‐4, Toronto Research Chemicals Inc.) stock solution with nominal concentration of 30 g/L was prepared in ethanol (95%, Solveco, analytical grade) and stored in darkness at 4℃. At each spiking event, 5 ml of stock solution was serially diluted in MilliQ water, and aquaria were spiked with 0.1 L to obtain nominal concentrations of 0, 1, or 100 µg/L (e.g., Weinberger & Klaper, 2014). Solvent concentrations were at least an order of magnitude lower than recommended for aquatic organisms (Hutchinson et al., 2006). On day zero of the experiment, we added FLX and pike according to treatments. The experiment was initiated the day after (day 1; 23 June 2015), when six crucian carp were introduced into each aquarium. Crucian carp were fed fish pellets and frozen shrimp mix (5% of fish weight) five times weekly. Pike in predator treatments were fed two crucian carp weekly. The feeding of pike was done in the experimental tank so that experimental subjects could detect the release of chemical alarm substances from the consumed conspecifics. Every 15 days (starting 7 July 2015), approximately 75% of the aquaria water was replaced and water re‐spiked with FLX to maintain exposure concentrations. Also, we sacrificed one fish from each aquarium on 7 July for another study, reducing replicate density from six to five fish. Fish that died during the experiment (*n* = 11) were immediately replaced with reserve fish, but only original individuals were used in the analyses (after 178 days, 23 June – 18 December 2015; *n* = 109).

### 2.3 Comparing predicted versus measured plasma concentrations

Plasma concentrations (*Fish_plasma_*) of fluoxetine were predicted by the model developed by (Huggett et al., 2003), derived from the plasma bioconcentration model by Fitzsimmons et al. (2001). Predictions were first made to select nominal water concentrations. The LOW water concentration aimed to result in plasma concentrations below human therapeutic concentrations (*C_max_*) for fluoxetine (and also in the same order of magnitude as concentration detected in the environment). The high concentrations aimed to result in plasma concentrations well above *C_max_*. These predictions were then compared with modeled plasma concentrations using measured water concentrations of fluoxetine and finally with measured plasma concentrations of fluoxetine.

The human therapeutic plasma concentration was defined as either 50 µg/L or 300 µg/L (Sumpter et al., 2014) each of which was used to calculate effect ratios (*ERs*).

$$ER=C_{\max}/Fish_{\mathrm{plasma}}$$

$$Fish_{\mathrm{plasma}}=EC\times P_{Blood:water}$$

$$LogP_{Blood:water}=0.73\times LogP_{oct:water}‐0.88.$$

where *EC* is the nominal or measured water concentration of fluoxetine (µg/L), *P_Blood:water_* is the partitioning coefficient for fluoxetine between fish blood and water, and *P_oct:water_* is the partitioning coefficient for fluoxetine between octanol and water. Log*P_oct:water_* was defined as 3.4 which is the geometric mean of 9 studies with measured coefficients (refs in Oakes et al., 2010, and Nakamura et al., 2008).

### 2.4 Measurement of FLX and norFLX concentrations

Our FLX treatments were confirmed measuring FLX in both water and crucian carp blood plasma. For initial water concentrations, we collected water (300 ml water in a 500‐ml Heraco glass jar) from 50% (random) of the replicates of all treatments on day 0, after stirring the aquarium water. To assess the fate of FLX in the water phase, we sampled water from a randomly selected “No predator +High FLX” aquaria eight times over 10 days after spiking. (Figure A1). Experimental half‐life, *t*_1/2_ (d) of fluoxetine in the water phase was calculated to 8.9 days (90% CI 5–46 days, Figure A1) assuming first‐order kinetics as:

$$\ln\left[C\right]=a\left(t\right)+b.$$

$$k_{d}=\left|a\right|$$

$$t_{1/2}=\frac{\ln\left(2\right)}{k_{d}}$$

Here, *C* is the measured concentration of fluoxetine in the water phase of the treatment, *t* is time after spiking of aquaria (d), and *k_d_* is the rate constant (d^‐1^).

Further, we collected blood samples from two fish per aquarium (*n* = 48) at the end of the experimental period (18 December) to measure blood concentrations of FLX and its biologically active metabolite norFLX. We followed the analytical protocols of Lindberg et al. (2014) for water samples and McCallum, Krutzelmann, et al. (2017) for blood plasma. In short, water samples (3 ml) were filtered through a 0.45 μm membrane filter (MF, Millipore, Sundbyberg, Sweden) and 5 ng of deuterated fluoxetine was added as internal and surrogate standard. Plasma samples (100 µl) were pretreated by adding 5 ng of deuterated fluoxetine and methanol with 0.1% formic acid (100 µl). All samples were frozen at −18℃ for one hour, thawed, and 200 µl of water (with 0.1% formic acid) was added. Samples were analyzed after being centrifuged at 17,500 *g* for 10 min. Both water and plasma samples were analyzed using a system with a triple‐stage quadrupole mass spectrometer (Quantum Ultra EMR (Thermo Fisher Scientific) coupled with a liquid chromatographic pump (Accela, Thermo Fisher Scientific) and an autosampler (PAL HTC, CTC Analytics AG, Zwingen, Switzerland). This was done to measure the actual concentration of fluoxetine and its biologically active metabolite norfluoxetine and the specific settings for the multiple reaction monitoring of norfluoxetine was precursor ion 296, product ion 30,1, collision energy 43, and tube lens at 50 V.

pH, oxygen, and conductivity were measured in all experimental aquaria at three time points (Tables A3‐A5).

### 2.5 Behavior

We quantified boldness, a key behavioral trait, of individual crucian carp at three different time points: 1) prior to the experimental treatments, 2) after short‐term exposure (two weeks; 17 days; 18 July), and 3) after long‐term exposure (24 weeks; 171 days, 11 December) to investigate effects of treatments on prey behavior. Behavioral assays were conducted in aged, aerated water (without FLX) of similar temperature as in the treatment aquaria (water replaced and arenas always rinsed between trials). To reduce environmental disturbance, a tarpaulin tent sheltered the assay container during trials. We used an established refuge emergence protocol where the boldness score is defined as the latency to emerge from a refuge (Brown et al., 2007; Hulthén et al., 2014). The assay arena consisted of a circular PVC tank (total volume: 70 L, water volume: 30 L), lined with ScotchLite luminous tape for background contrast, and a gray PVC refuge box (28 × 20×20 cm). For each assay, one haphazardly chosen crucian carp was moved from an exposure tank to the refuge box, given 5 min to acclimatize, before a vertically sliding trapdoor was slowly raised via a remote pulley system. We observed refuge emergence behavior via a video camera (Logitech C920) centrally mounted above the tank and linked to a monitor placed outside the tent. Time taken to emerge (the whole body of the fish outside the box) was recorded to the nearest 1 s. Each trial lasted for 10 min, and nonemerging fish were given a ceiling value of 600 s.

### 2.6 Morphology

Morphological changes were measured using landmark‐based geometric morphometric methods. At the end of the experiment, fish were netted from their home tanks, anaesthetized with benzocaine (Sigma‐Aldrich, Ethyl p‐aminobenzoate), laterally placed on a white foam board, and digitally photographed. A ruler was included in each image for scale calibration. From the images, we measured standard length (SL) and digitized 10 homologous landmarks (Figure A2) using tpsDig2 (Rohlf, 2017a). We performed Generalized Procrustes Analysis to scale, rotate, and superimpose landmarks (removing isometric size effects and all other nonshape variation). We then used tpsRelw (Rohlf, 2019) to extract centroid size (the square root of the sum of squared distances from landmarks to their centroid) as an estimate of body size, and the 16 partial warps and uniform components as geometric morphometric descriptors of body shape.

### 2.7 Statistical analyses

FLX concentrations (ng/ml, log_10_‐transformed for normality) in the water among treatments were analyzed using ANOVA with factors FLX and Predator treatments, including their interaction. Analyses of FLX and norFLX concentrations in crucian carp blood plasma were conducted using nested mixed‐model ANOVA for the separate variables, including random factor Tank nested within the FLX×Predator interaction term. Significant interaction terms were evaluated with nine planned contrasts to test for effects of Predator treatments within each of the FLX treatments, and vice versa. We adjusted significance levels to maintain a false discovery rate (FDR) of 5% (Benjamini & Hochberg, 1995).

109 individuals were tested for short‐ and long‐term effects of experimental treatments on boldness, evaluating for calculated change in boldness from a pre‐experiment measurement to two weeks (17 days) and to 24 weeks (171 days) of experimental exposure, respectively (latter behavioral score minus the pre‐experiment score for individuals). These measurements were evaluated in separate nested mixed‐model ANOVAs as described above. We initially included log_10_‐transformed standard length as a covariate, to control for potential effects of body size, but excluded it due to nonsignificance (both *p* > .55).

Effects of treatments on body shape were tested using nested mixed‐model multivariate analysis of covariance (MANCOVA) (for details see Hassell et al., 2012; Heinen‐Kay & Langerhans, 2013; Riesch et al., 2013). The 16 partial warps and uniform components served as dependent variables, centroid size as a covariate controlling for multivariate allometry, and FLX and Predator treatments, with their interaction, along with random factor Tank nested within the FLX×Predator interaction term as factors. We evaluated the relative importance of model terms using the multivariate effect size estimate of Wilks's partial η^2^ (e.g., Langerhans & DeWitt, 2004). We used two approaches to visualize morphological differences among treatments. First, to explicitly examine effects of experimental treatments on size‐adjusted body shape, we calculated the divergence vectors (d) derived from the terms of interest in the MANCOVA and visualized variation along these axes using thin‐plate spline deformations in tpsRegr (Rohlf, 2016). These divergence vectors accurately describe axes of greatest variation separating treatment groups, while controlling for other terms in the model (details in Langerhans, 2009, Langerhans & Makowicz, 2009). Second, to describe the overall differences in body shape among treatment groups, we calculated the landmark consensus within each treatment combination and the Procrustes distance for the nine relevant comparisons using tpsSmall (Rohlf, 2017b). Procrustes distance represents the standard metric of shape differences in geometric morphometrics (e.g., Bookstein, 1996) and is closely approximated by Euclidean distance between landmarks after generalized Procrustes analysis (Zelditch et al., 2004). All statistical analyses were performed using the software SAS, Institute, Cary, NC.

## 3 RESULTS

### 3.1 Water and plasma concentrations of FLX and norFLX

Overall, we confirmed that our administration of FLX was successful, as we detected around two orders of magnitude differences in FLX concentrations in water between the Low FLX and High FLX treatments. Measured water concentrations of FLX (after spiking) were lower than nominal targets (Table A1). FLX concentrations in treatments with a predator were 4%–6% of nominal (geometric means 0.056 and 3.6 µg/L, in Low FLX and High FLX, respectively), and 18%–21% of the nominal from the treatments without a predator (geometric means 0.21 and 18 µg/L, in Low FLX and High FLX, respectively). As expected, we observed strong differences in FLX concentrations between the FLX treatments (Table 1), with the FLX concentrations in the Low FLX treatment 70 to 90 times lower than in the High FLX treatment. FLX treatments without predator had higher water concentration of FLX than FLX treatments with predator, albeit marginally nonsignificant (*p* = .052) and with no interaction between treatments (Table 1).

**TABLE 1:** Results of statistical models examining effects of fluoxetine (FLX) and the presence of a pike (Predator) on environmental and biotic factors of interest

Dependent variable	Factor	*df*	*F*	*p*
Water concentrations of fluoxetine∼	FLX	2, 6	167	<.001***
	Predator	1, 6	5.83	.052
	FLX × Predator	2, 6	1.51	.294
Plasma concentrations of fluoxetine∼	FLX	2, 18	4,113.2	<.001***
	Predator	1, 18	1.9	.185
	FLX × Predator	2, 18	4.52	.026*
Plasma concentrations of norfluoxetine∼	FLX	2, 18	673.91	<.001***
	Predator	1, 18	0.53	.475
	FLX × Predator	2, 18	0.14	.868
ΔBoldness at two weeks∼	FLX	1, 17.31	6.15	.010**
	Predator	1, 17.33	0.3	.589
	FLX × Predator	1, 17.33	1.34	.288
ΔBoldness at twelve weeks∼	FLX	2, 17.63	0.06	.945
	Predator	1, 17.65	0	.949
	FLX × Predator	2, 17.63	0.32	0.73
Morphology∼	Centroid size	15, 644	2.48	.001**
	FLX	30, 902	3.29	<.001***
	Predator	15, 644	24.74	<.001***
	FLX × Predator	30, 902	1.6	.022*

* *p* < .05, **p < .01, ***p < .001.

**FIGURE 1:**
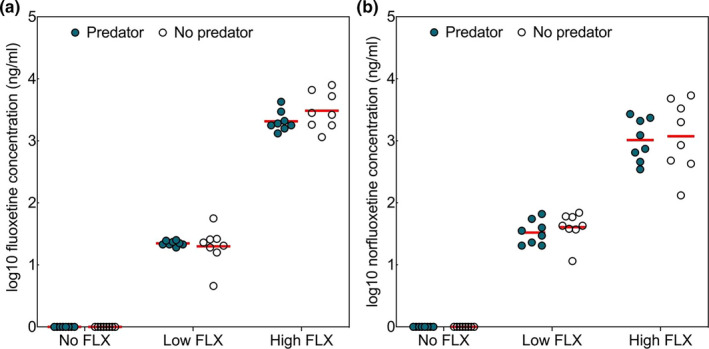
Variation in blood plasma concentrations of (a) fluoxetine and (b) norfluoxetine among crucian carp from six different treatment combinations of (pike) predator presence and fluoxetine concentration (0, 1, and 100 μg/L). Red lines denote the group means

We also found strong differences between FLX treatments in the concentration of FLX and norFLX in crucian carp blood plasma (Figure 1a,b, Table 1). Blood plasma concentrations of FLX were below the limit of quantification in the No FLX treatment and 90–150 times lower in the Low FLX treatment than the High FLX treatment (Table A2). Although most of the variation in FLX concentration in blood plasma was caused by differences between FLX treatments, we did detect a significant interaction term between Predation and FLX treatments (Table 1). We found clear differences between all FLX treatments (planned contrasts) whether in the absence or presence of pike (all *p* < .001), no effect of the Predator treatment in No FLX (*p* = 1.0) or Low FLX treatment (*p* = .476), but a slightly elevated FLX concentration in the absence of a predator in the High FLX treatment (*p* = .006, Figure 1a). Blood plasma concentrations of norFLX showed no influence of the Predator treatment (Table 1) and were 33–40 times lower in the Low FLX treatments (No predator +Low FLX = geometric means 33 ng/ml, Predator +Low FLX = 40 ng/ml, No predator +High FLX = 1,000 ng/ml and Predator +High FLX = 1,200 ng/ml), Figure 1b.

### 3.2 Behavior

**FIGURE 2:**
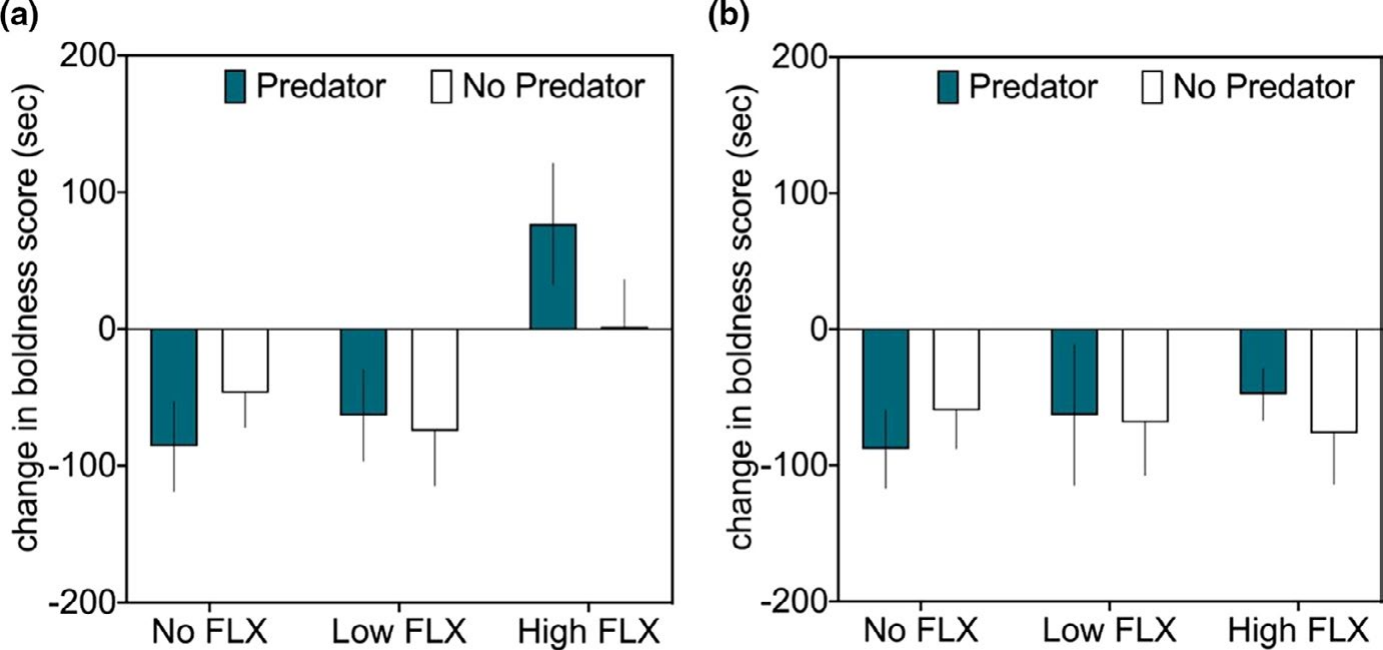
Behavioral response (mean ± 1 SE) of individual crucian carp quantified and illustrated as the change in boldness score from pre‐experimental values to scores after (a) two weeks and (b) 24 weeks of treatment exposure. Boldness score refers to the time (in sec) it took for each individual fish to leave the experimental refuge box, that is, positive values indicate shyer fish

We found a strong effect of short‐term exposure to FLX treatment on boldness, but neither predator treatment nor the interaction was significant (Table 1). Fish took longer to emerge from the refuge in the High FLX treatment after 17 days, but this effect had vanished after six months when all fish showed similar boldness levels (Figure 2).

### 3.3 Morphology

**FIGURE 3:**
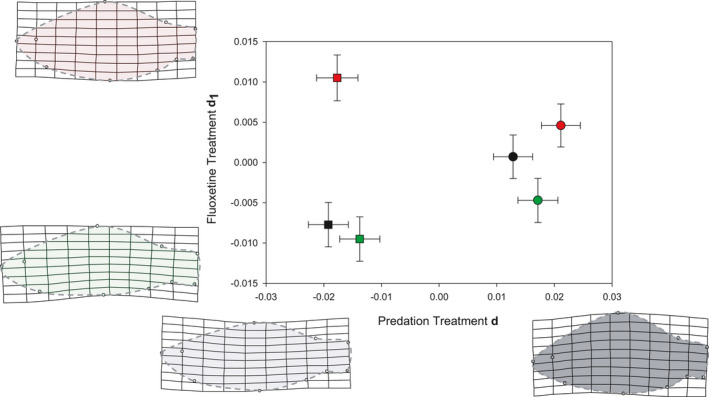
Thin‐plate spline transformation grids showing the body shape variation of crucian carp described by each axis, with means and standard errors for each treatment group. Divergence vectors (d) were calculated from the terms of interest in the MANCOVA. Observed range of variation depicted (no magnification). Dashed lines illustrate the fish body outline as an aid for visual interpretation. Squares: Predator absent, circles: Predator present, black: No FLX, green: Low FLX, red: High FLX

**FIGURE 4:**
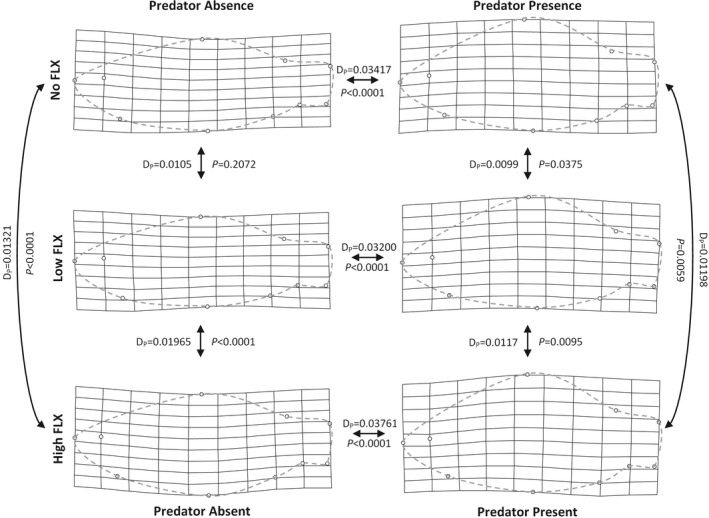
Thin‐plate spline transformation grids illustrating the landmark consensus for each treatment group relative to the overall mean body shape (magnification 2×). The Procrustes Distances (*D*_P_) and adjusted *p*‐values of group differences are provided by the arrows. Dashed lines illustrate the fish body outline as an aid for visual interpretation

We found significant effects of all model terms on body shape in our mixed‐model MANCOVA (Table 1). Based on our multivariate estimate of effect size, the Predator treatment clearly had the strongest effect on body shape (partial variance explained: Centroid size = 39.11%, FLX = 44.79%, Predator = 80.36%, FLX × Predator = 36.14%). Crucian carp exposed to a pike predator developed deeper bodies characterized by a strong dorsal shift in the dorsal‐fin insertion landmark (Figure 3). This effect was largely independent of FLX treatment, with marked differences between Predator treatments within all three FLX treatments (all *p* < .0001, Figure 4). However, the interaction term revealed that the effect of FLX was context‐dependent. In the presence of pike, body shape differed between all FLX treatments, although not especially strong in magnitude (Figures 3, 4). In the absence of a predator, however, the Low FLX treatment had no discernible effect on body shape, while the High FLX treatment had a pronounced effect as it induced an increased body depth (Figures 3, 4). In contrast to the effect of Predator, the FLX effect mainly resulted from a ventral shift of the pelvic fin insertion landmark.

## 4 DISCUSSION

Exposure to FLX, a selective serotonin reuptake inhibitor, affects disparate antipredator traits in crucian carp. First, we found that exposure to high concentrations of FLX affected the propensity to take risks (boldness). After short‐term exposure to a high concentration of FLX, fish became relatively shyer whereas unexposed fish and individuals exposed to a low concentration shifted toward increased boldness. Intriguingly, the initial, strong effect of high FLX disappeared after six months. Second, we found that FLX exposure altered the expression of the inducible morphological defense. The effect of FLX on body shape was context‐dependent, where the high FLX treatment strongly influenced morphology of fish regardless of the prevailing risk of predation, whereas the low FLX treatment influenced the body shape in the presence but not in the absence of a predator.

Water concentrations of FLX were lower than the nominal targets, especially for treatments with pike, but differences between High and Low FLX treatments were consistently close to the targeted two orders of magnitude. Blood plasma levels of FLX were also around two orders of magnitude higher in High FLX treatments, and here highest in the No predator +High FLX treatment. A potential explanation to the marginally lower water concentrations in predator treatments is uptake and bioaccumulation of FLX in pike. Exposure and bioaccumulation of FLX may alter pike behavior affecting predator–prey interaction strengths and perceived predation risk in crucian carp. However, we have shown that chemical cues are enough to trigger a behavioral and morphological response in crucian carp (Brönmark & Pettersson, 1994; Höglund et al., 2005; Pettersson et al., 2000), and hence, it is unlikely that an altered behavior in pike (changing visual cues) would have had any effect on the overall antipredator response in crucian carp. Plasma level in the High FLX treatments exceeded human therapeutic levels by at least an order of magnitude, and hence, we expect that we achieved the intended serotonergic effect on the stress axis in the High FLX. However, levels were 2 to 10 times lower than human therapeutic levels in the Low FLX treatment.

The temporary behavioral shift of FLX toward a shyer phenotype matched our a priori prediction and adds to the growing evidence that exposure to FLX can impact key fitness‐related behaviors owing to the causal link between the serotonergic system and the stress axis (Wong & Bymaster, 1995, Lowry, 2002, Gesto et al., 2013, Vindas et al., 2017, Hesketh et al., 2005). FLX is known to affect disparate behaviors in vertebrates, including activity (Barry, 2013), boldness (Winberg & Thörnqvist, 2016), shoaling (Giacomini et al., 2016), aggression (McCallum, Bose, et al., 2017), prey capture (Gaworecki & Klaine, 2008), feeding rate, and growth (Dorelle et al., 2020). However, boldness in crucian carp was only affected by High FLX and not by Low FLX, the concentration that fish may encounter in the wild. This is in contrast to earlier studies where, for example, crabs reared in presence of a predator showed an increase in foraging and activity along with a more aggressive personality style when exposed to environmentally relevant concentrations of FLX (Peters et al., 2017). However, bioaccumulation of FLX differs between taxa (Boström et al., 2017; Lagesson et al., 2016) and drug targets and affinity may also vary, making the read‐across hypothesis less applicable outside its intended domain. As predicted by the fish plasma model, the low FLX treatment indicated plasma levels in crucian carp just below those of expected therapeutic effects.

To date, few studies, if any, have examined the effects of long‐term exposure to psychoactive drugs on antipredator behaviors in vertebrate prey. In general, acute and initial exposure to SSRIs is known to cause contrasting effects compared to long‐term chronic exposure. For example, short‐term exposure has been linked to anxiety‐like behavior with high stress sensitivity, whereas long‐term exposure often results in an opposite effect due to desensitizing of the stress axis (Herculano & Maximino, 2014; Jensen et al., 1999; Lepage et al., 2005). Organisms exposed to SSRI’s may also build up tolerance, which may explain why pharmacological effects attenuate over time. However, environmental concentrations of pharmaceuticals may fluctuate dramatically over time and across seasons, which could hamper pollution‐induced tolerance (Vossen et al., 2020). Still, the temporal differences in the effect of FLX on behavior in this study clearly show the importance of running experiments on ecologically relevant time scales.

Previous studies have shown that exposure to predator cues result in a deeper body morphology in crucian carp (Brönmark & Miner, 1992; Brönmark & Pettersson, 1994; Johansson & Andersson, 2009), and although the proximate, physiological mechanisms behind the change in morphology are currently unknown, recent studies have suggested that the expression of inducible defense traits may be linked to changes in neuroendocrine pathways coupled to the physiological stress response (Hossie et al., 2010; Maher et al., 2013; Vinterstare, Hulthén, Nilsson, Nilsson Sköld & Brönmark, 2020). Due to the stress‐dampening effects of SSRIs, we initially expected FLX exposure to cause a reduction in the morphological response to perceived predation risk, such that predator exposed fish also exposed to FLX would show less expression of the defense compared to fish not exposed to FLX. However, exposure to pike induced a strong and highly significant change in crucian carp morphology resulting in much deeper bodies in the presence of predators. While the magnitude of the predator‐induced morphological shift was relatively unaltered by FLX, the resulting predator‐induced morphology differed among FLX treatments, indicating FLX exposure might affect the vulnerability of crucian carp to predation.

In contrast, in the absence of predators, Low FLX had no effect on body shape, whereas High FLX affected morphology. However, while the presence of a predatory pike caused a change dorsally, FLX exposure increased fish body depth ventrally, that is, fish became chubbier when exposed to FLX. The contrasting effects in presence/absence of pike might be related to changes in activity. Crucian carp reduce their activity in the presence of predators (Pettersson et al., 2000; Vinterstare, Hulthén, Nilsson, Nilsson, & Brönmark et al., 2020) and earlier study has linked the regulation of the morphological defense expression in crucian carp to differences in activity levels observed with or without predators (Johansson & Andersson, 2009); energy saved by being less active could be allocated to growth. However, our morphometric data clearly show that predator exposure results in increased body depth via dorsal and not ventral change, the latter being expected from reduced activity levels and evident among FLX exposed crucian carp. Thus, FLX exposure appears to result in relatively heavier crucian carp with ventrally deepened bodies, likely a nonadaptive body shape as it should reduce fitness due to increased energetic costs of transport (Pettersson & Brönmark, 1997, 1999).

## 5 CONCLUSION

In conclusion, FLX and its biologically active metabolite norFLX affected predator‐driven changes in behavior and morphology of crucian carp. The effect on behavior diminished after long‐term exposure, whereas the effect on morphology was in an unpredicted direction. The effect of FLX further suggests that serotonin plays a key role in the stress response by modulating the HPA/HPI axis and that such changes in stress physiology are of considerable interest when it comes to our understanding of the underlying mechanisms that regulate inducible antipredator responses. Hence, anthropogenically derived FLX has the potential to disrupt phenotypes of aquatic taxa, including antipredator responses, and consequentially alter ecological interactions and impact fitness.

## CONFLICT OF INTEREST

We declare no conflict of interest.

## AUTHOR CONTRIBUTION

**Jerker Vinterstare:** Data curation (lead); Formal analysis (equal); Investigation (equal); Methodology (supporting); Validation (equal); Visualization (equal); Writing‐original draft (lead); Writing‐review & editing (equal). **Christer Brönmark:** Conceptualization (equal); Funding acquisition (lead); Investigation (equal); Resources (lead); Supervision (lead); Writing‐original draft (lead); Writing‐review & editing (equal). **P. Anders Nilsson:** Conceptualization (supporting); Formal analysis (supporting); Writing‐review & editing (equal). **R. Brian Langerhans:** Data curation (equal); Formal analysis (lead); Software (lead); Validation (equal); Visualization (lead); Writing‐review & editing (equal). **Olof Berglund:** Conceptualization (equal); Investigation (equal); Methodology (equal); Validation (equal); Writing‐review & editing (equal). **Jennie Örjes:** Investigation (equal); Methodology (equal); Writing‐review & editing (equal). **Tomas Brodin:** Validation (equal); Writing‐review & editing (equal). **Jerker Fick:** Investigation (equal); Methodology (equal); Validation (equal); Writing‐review & editing (equal). **Kaj Hulthén:** Conceptualization (equal); Data curation (equal); Formal analysis (equal); Investigation (equal); Methodology (equal); Validation (equal); Writing‐review & editing (equal).

## Supporting information

Supplementary Material

## Data Availability

Vinterstare, Jerker (2021), Data for Antipredator phenotype in crucian carp altered by a psychoactive drug, Dryad, Dataset, https://doi.org/10.5061/dryad.2ngf1vhnh
