# Supplementary material for: Antipredator phenotype in crucian carp altered by a psychoactive drug
Source: Ecol Evol. 2021 Jun 14;11(14):9435–46. doi: 10.1002/ece3.7762 (PMC8293787; doi:10.1002/ece3.7762)
Supplement: Supplementary file 1 — Supplementary Material [file ECE3-11-9435-s001.docx]

**Appendix for: Anti-predator phenotype in crucian carp altered by a psychoactive drug**

**Table A1:** Geometric means (min - max) of measured concentrations (µg L^-1^) of fluoxetine and norfluoxetine in water and in crucian carp (*Carassius carassius*) blood plasma among the different experimental treatments. < LOQ denotes below limit of quantification.

|  | **No FLX**  **No Pred** | **No FLX**  **Pred** | **Low FLX**  **No Pred** | **Low FLX**  **Pred** | **High FLX**  **No Pred** | **High FLX**  **Pred** |
| --- | --- | --- | --- | --- | --- | --- |
| **Water (*n per replicate* = 2)** |  |  |  |  |  |  |
| Fluoxetine | <LOQ | <LOQ | 0.21 | 0.056 | 18 | 3.6 |
|  |  |  | (0.20 - 0.21) | (0.025 - 0.13) | (8.2 - 41) | (2.4-5.4) |
| Norfluoxetine | <LOQ | <LOQ | <LOQ | <LOQ | 0.070 | 0.046 |
|  |  |  |  |  | (<LOQ - 0.070) | (<LOQ - 0.046) |
| **Blood Plasma (*n per replicate* = 8)** |  |  |  |  |  |  |
| Fluoxetine | <LOQ | <LOQ | 20 | 22 | 3100 | 2100 |
|  |  | <LOQ | (4.6 - 56) | (19 - 25) | (1100 - 8000) | (1300 - 4300) |
| Norfluoxetine | <LOQ | <LOQ | 40 | 33 | 1200 | 1000 |
|  |  |  | (12 - 69) | (20 - 65) | (130 - 5400) | (350 - 2700) |

**Table A2.** Modeled and measured fluoxetine plasma concentrations (*Fish_plasma_*) in crucian carp (*Carassius carassius*) after 147 days in Low and High water exposure (*EC*) treatments, and associated effect ratios (*ER*).

|  | ***EC* (µg L^-1^)** | ***Fish_plasma_* (µg L^-1^)** | ***ER^a^*** |
| --- | --- | --- | --- |
| **Modeled LOW nominal *EC*** | 1*^b^* | 38 | 1.3 - 7.8 |
| **Modeled LOW measured *EC*** | 0.11*^c^* | 4.1 | 12 - 73 |
| **Measured LOW** | - | 21*^f^* | 2.4 - 14 |
| **Modeled HIGH nominal *EC*** | 100*^b^* | 3800 | 0.013 - 0.078 |
| **Modeled HIGH measured *EC*** | 8.2*^d^* | 310 | 0.16 - 0.96 |
| **Measured HIGH** | - | 2500*^g^* | 0.02 - 0.12 |

*^a^*Range using *C*_max_ 50 - 300 µg L^-1^

*^b^*Nominal water concentration

*^c^*Geometric mean of measured water concentrations of fluoxetine in Low treatments (with and without predator, *n* = 4), after spiking.

*^d^*Geometric mean of measured water concentrations of fluoxetine in High treatments (with and without predator, *n* = 4), after spiking.

*^f^*Geometric mean of measured blood plasma concentrations in Crucian carp in Low treatments (with and without predator, *n*=16), at day 147.

*^g^*Geometric mean of measured blood plasma concentrations in Crucian carp in High treatments (with and without predator, *n*=16), at day 147.

**Figure A1.** Estimation of experimental fluoxetine *t*_1/2_ in water by linear regression analysis of ln*C* =a(*t*)+b. The slope of the regression ln*C* = - 0.078(*t*) + 3.7, *r^2^* = 0.63, was used to calculate fluoxetine half-life to 8.9 days in the treatments with High FLX no predator. In the treatments with High FLX predator, half-life was not determined due to poor model fit (slope not different from zero).

**Figure A2**. Illustration of the locations of the ten fixed landmarks used to examine the effects of fluoxetine and predator exposure on crucian carp morphology.


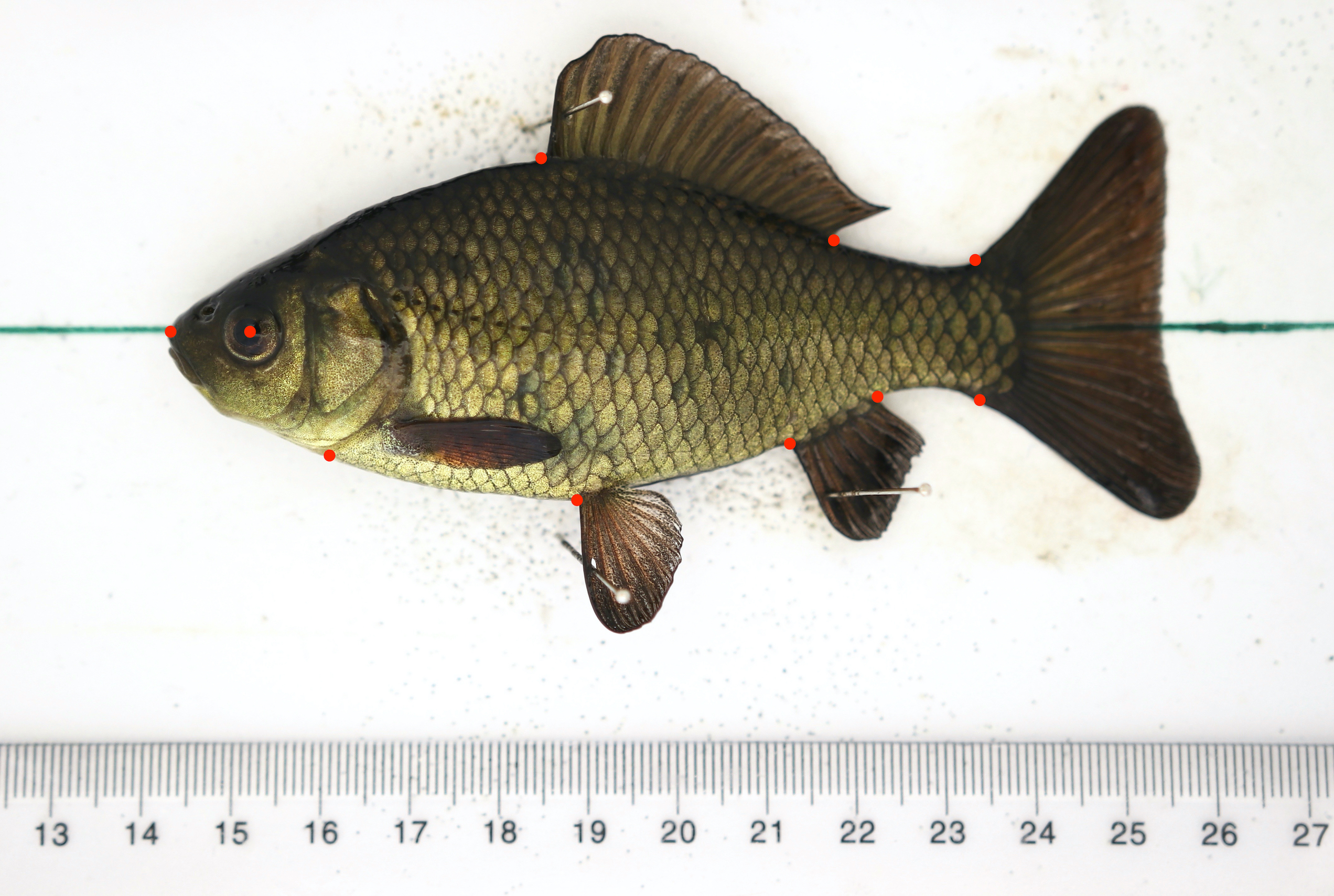


**Table A3.** pH values from each experimental unit measured over the course of the first spike of fluoxetine i.e. from day 1 to day 15, when 75% of the water was changed and water re-spiked with fluoxetine to maintain exposure concentrations

| **Tank** | **Treatment** | **pH 23 June** | **pH 1 July** | **pH 8 July** |
| --- | --- | --- | --- | --- |
| 1 | No FLX + Predator | 7.6 | 7.4 | 7.6 |
| 2 | High FLX + No predator | 7.5 | 7.8 | 7.6 |
| 3 | High FLX + Predator | 7.5 | 7.3 | 7.5 |
| 4 | Low FLX + Predator | 7.6 | 7.5 | 7.7 |
| 5 | No FLX + No predator | 7.6 | 7.7 | 7.6 |
| 6 | No FLX + No predator | 7.5 | 7.8 | 7.6 |
| 7 | Low FLX + No predator | 7.6 | 7.8 | 7.7 |
| 8 | Low FLX + Predator | 7.6 | 7.3 | 7.5 |
| 9 | No FLX + Predator | 7.5 | 7.5 | 7.6 |
| 10 | High FLX + Predator | 7.5 | 7.4 | 7.5 |
| 11 | Low FLX + Predator | 7.6 | 7.4 | 7.6 |
| 12 | High FLX + Predator | 7.6 | 7.4 | 7.6 |
| 13 | No FLX + Predator | 7.5 | 7.3 | 7.6 |
| 14 | Low FLX + No predator | 7.7 | 7.9 | 7.8 |
| 15 | High FLX + No predator | 7.6 | 7.9 | 7.8 |
| 16 | No FLX + Predator | 7.5 | 7.2 | 7.4 |
| 17 | No FLX + No predator | 7.6 | 7.8 | 7.7 |
| 18 | High FLX + Predator | 7.4 | 7.3 | 7.5 |
| 19 | No FLX + No predator | 7.5 | 7.7 | 7.6 |
| 20 | Low FLX + No predator | 7.7 | 7.8 | 7.8 |
| 21 | Low FLX + Predator | 7.6 | 7.5 | 7.7 |
| 22 | Low FLX + No predator | 7.6 | 7.8 | 7.7 |
| 23 | High FLX + No predator | 7.6 | 7.9 | 7.7 |
| 24 | High FLX + No predator | 7.8 | 7.9 | 7.8 |

**Table A4**: Oxygen levels from each experimental unit measured over the course of the first spike of fluoxetine i.e. from day 1 to day 15, when 75% of the water was changed and water re-spiked with fluoxetine to maintain exposure concentrations.

| **Tank** | **Treatment** | **O_2_ (mg/l) 24 June** | **O_2_ (mg/l) 2 July** | **O_2_ (mg/l) 8 July** |
| --- | --- | --- | --- | --- |
| 1 | No FLX + Predator | 8.5 | 8.6 | 8.6 |
| 2 | High FLX + No predator | 8.4 | 8.6 | 8.3 |
| 3 | High FLX + Predator | 7.9 | 7.9 | 8.1 |
| 4 | Low FLX + Predator | 8.5 | 8.6 | 8.5 |
| 5 | No FLX + No predator | 8.6 | 8.9 | 8.4 |
| 6 | No FLX + No predator | 8.6 | 8.7 | 8.6 |
| 7 | Low FLX + No predator | 8.6 | 8.6 | 8.6 |
| 8 | Low FLX + Predator | 8.1 | 8.2 | 8.4 |
| 9 | No FLX + Predator | 8.6 | 8.7 | 8.6 |
| 10 | High FLX + Predator | 8.5 | 8.5 | 8.4 |
| 11 | Low FLX + Predator | 8.5 | 8.4 | 8.4 |
| 12 | High FLX + Predator | 8.5 | 8.4 | 8.5 |
| 13 | No FLX + Predator | 8.4 | 8.3 | 8.5 |
| 14 | Low FLX + No predator | 8.8 | 8.8 | 8.8 |
| 15 | High FLX + No predator | 8.7 | 8.8 | 8.6 |
| 16 | No FLX + Predator | 8 | 7.9 | 7.9 |
| 17 | No FLX + No predator | 8.7 | 8.8 | 8.7 |
| 18 | High FLX + Predator | 7.7 | 7.7 | 7.8 |
| 19 | No FLX + No predator | 8.6 | 8.7 | 8.6 |
| 20 | Low FLX + No predator | 8.7 | 8.7 | 8.7 |
| 21 | Low FLX + Predator | 8.4 | 8.7 | 8.8 |
| 22 | Low FLX + No predator | 8.7 | 8.7 | 8.8 |
| 23 | High FLX + No predator | 8.6 | 8.8 | 8.6 |
| 24 | High FLX + No predator | 8.8 | 8.8 | 8.8 |

**Table A5**. Conductivity levels from each experimental unit measured over the course of the first spike of fluoxetine i.e. from day 1 to day 15, when 75% of the water was changed and water re-spiked with fluoxetine to maintain exposure concentrations.

| **Tank** | **Treatment** | **Conduc. (uS) 23 June** | **Conduc. (uS) 1 July** | **Conduc. (uS) 8 July** |
| --- | --- | --- | --- | --- |
| 1 | No FLX + Predator | 197 | 215 | 206 |
| 2 | High FLX + No predator | 184 | 206 | 197.4 |
| 3 | High FLX + Predator | 197 | 220 | 202 |
| 4 | Low FLX + Predator | 196 | 216 | 197 |
| 5 | No FLX + No predator | 186 | 208 | 196.5 |
| 6 | No FLX + No predator | 189 | 208 | 199.1 |
| 7 | Low FLX + No predator | 188 | 202 | 197.7 |
| 8 | Low FLX + Predator | 201 | 221 | 204 |
| 9 | No FLX + Predator | 192 | 225 | 210 |
| 10 | High FLX + Predator | 190 | 206 | 197 |
| 11 | Low FLX + Predator | 193 | 222 | 202 |
| 12 | High FLX + Predator | 191 | 220 | 205 |
| 13 | No FLX + Predator | 190 | 214 | 197 |
| 14 | Low FLX + No predator | 182 | 206 | 194 |
| 15 | High FLX + No predator | 186 | 204 | 197 |
| 16 | No FLX + Predator | 197 | 220 | 207 |
| 17 | No FLX + No predator | 187 | 210 | 199.8 |
| 18 | High FLX + Predator | 193 | 218 | 193 |
| 19 | No FLX + No predator | 228 | 257 | 216 |
| 20 | Low FLX + No predator | 189 | 209 | 199 |
| 21 | Low FLX + Predator | 184 | 207 | 197 |
| 22 | Low FLX + No predator | 190 | 207 | 199.6 |
| 23 | High FLX + No predator | 181 | 203 | 198.4 |
| 24 | High FLX + No predator | 183 | 205 | 195.5 |
